# Conversational facial signals combine into compositional meanings that change the interpretation of speaker intentions

**DOI:** 10.1038/s41598-024-52589-0

**Published:** 2024-01-27

**Authors:** James P. Trujillo, Judith Holler

**Affiliations:** 1 Max Planck Institute for Psycholinguistics, Nijmegen, The Netherlands; 2 Donders Institute for Brain, Cognition, and Behaviour, Nijmegen, The Netherlands

**Keywords:** Human behaviour, Social evolution

## Abstract

Human language is extremely versatile, combining a limited set of signals in an unlimited number of ways. However, it is unknown whether conversational visual signals feed into the composite utterances with which speakers communicate their intentions. We assessed whether different combinations of visual signals lead to different intent interpretations of the same spoken utterance. Participants viewed a virtual avatar uttering spoken questions while producing single visual signals (i.e., head turn, head tilt, eyebrow raise) or combinations of these signals. After each video, participants classified the communicative intention behind the question. We found that composite utterances combining several visual signals conveyed different meaning compared to utterances accompanied by the single visual signals. However, responses to combinations of signals were more similar to the responses to related, rather than unrelated, individual signals, indicating a consistent influence of the individual visual signals on the whole. This study therefore provides first evidence for compositional, non-additive (i.e., Gestalt-like) perception of multimodal language.

## Introduction

One of the fundamental aspects of human behavior is communication, such as the way that we use language to coordinate, interact, and connect with one another. Indeed, the complexity and flexibility of human language is thought to be a key difference between humans and other mammals^1^. Part of what makes language so versatile is that we can combine a limited set of signals (e.g., vocal sounds, or hand movements) in an unlimited number of ways. Understanding how human language evolved, and how it is produced and perceived, therefore rests heavily on understanding how the combining of individual parts, such as words, leads to the unified meaning of a larger unit, such as an utterance. While there is much work discussing how words combine into more complex meaningful utterances in spoken or sign language, a fundamental yet unaddressed question is how complex meaning is derived in the case of multimodal utterances, involving not only speech but also visual signalling. This is because in face-to-face interaction, the very environment in which human language has evolved, facial expressions and other visual signals are ubiquitous and part of the process of conveying information, in both signed and spoken language exchanges. Answering this question is thus essential to fully understand how human language is used to create meaning.

Discussions on the compositional nature of language have emphasized how the meaning of a sentence comes from the combination of its constituent parts^1–7^. A similar case for compositionality has been made for sign languages, where meaning comes from the hands, head, face and body^8–12^. Particularly interesting when considering the evolution of language is that the gradual recruitment of additional articulators (e.g., the head, face, body) seems to positively correlate with the increase in grammatical complexity that is seen in emerging and developing sign languages^12^.

Despite making the important step of demonstrating the inherently compositional nature of human language, past accounts of compositionality have focused on language from a unimodal perspective, typically describing language as “speech or sign”^4,7, 13, 14^. This is in contrast to many recent accounts that point out the fundamentally multimodal nature of human language, which includes visual signals such as facial expressions, body posture, and manual gesture^15–20^, as well as work that already framed meaning in spoken language as arising from a composite of spoken and visual signals^21–24^. Moving investigations of the compositional nature of human language into this domain is a vital next step and facilitated by the insights from how visual signals combine in sign languages^12^.

In the absence of speech, visual signals have already been shown to exhibit compositional meaning. For example, using photos of athletes displaying various emotional responses, with participants interpreting the emotion based on a combination of bodily and facial cues^25^. Similarly, Liu and colleagues^26^ used virtual avatar faces to model a large range of facial expressions and showed that combinations of facial expression components (e.g., movement of eyebrows, cheeks, mouth) jointly signal both broad and specific emotions^26^. Along similar lines, Nölle and colleagues^27^ provided evidence that certain iconic facial features (e.g., eye widening, nostril flare) can signal specific intentions, such as rejection or acceptance. However, these studies focused on visual signals outside of language. How visual signals contribute to the pragmatic interpretation, or intention recognition, of a *multimodal* utterance combining speech and visual signals as they occur in conversational settings is an open question and the focus of this study.

There is also a recent work on how visual signals combine to form more complex meanings in non-human animals^28–32^. Although Kendon^33^ has suggested that language is a whole-body phenomenon, there is currently no empirical evidence for whether visual signals in human face-to-face communication contribute compositionally to conversational utterances.

Facial signalling is ubiquitous in conversation and an important carrier especially of pragmatic information (e.g., whether an utterance is meant ironically, directing the listener’s attention, signalling uncertainty about the factuality of a statement), and signals often combine with one another and with speech to convey communicative intentions^34–38^. However, facial expressions of emotions (outside of the context of language) have been described as discrete and holistic, and thus as contrasting with the compositional nature of (bodily) language^10,12, 25, 30^. Here, we propose that these two notions are not incompatible, at least when we consider conversational facial expressions and pragmatic meaning communication. This is because while individual facial signals may combine into more complex messages in a way that they should be considered compositional, the psychological processes underlying their interpretation into meaningful messages may closely follow Gestalt-psychological processes, in particular the notion that “the whole is more than the sum of its parts”—where the original actually translated ‘not the same’ as the sum of its parts^39^. This fits well with recent theoretical framings of human multimodal language as Gestalt-like^15,19, 40–43^. It also fits with the recent, more flexible notion of compositionality in the animal literature^28,30^. The traditional, linguistic notion of compositionality entails that individual units have stable meanings and then when those stable meanings are combined into more complex ones, the complex meaning is fully interpretable based on the meanings of the individual units (i.e. additive combination). However, moving beyond conventionalized meanings may open up meaning generation that extends to novel, non-additive interpretations, such as in a study which observed a facial expression combined with different arm gestures resulting in different responses in chimpanzees (and different to the responses following the individual visual signals), by Oña and colleagues^30^. In the light of the possibility of observing non-additive meaning resulting from the combination of visual signals, we can therefore consider compositional meaning also as a form of Gestalt meaning.

If there is indeed evidence for meaning being derived from inputs in such a Gestalt-like, non-additive way (i.e., the combination of signals creating meaning that is different from just adding the meaning of the components), this would be of direct relevance to how we conceptualise human language. First, because it would demonstrate that conversational facial signals contribute to the interpretation of utterance meaning, and second, because their specific combinations would lead to a difference in meaning attribution. This would fundamentally change the way that we must study language use, processing and language evolution. In particular, a non-additive, Gestalt-like relationship between the meaning of the whole and the meaning of the parts would have important consequences for our conceptualization of human language and studies that attempt to experimentally or analytically separate communicative behavior into isolated modalities.

### The current study

The current study provides a first test of whether the perception of human multimodal communicative signals is non-additive and Gestalt-like in nature. Direct evidence for the hypothesis that multimodal signals combine non-additively to form utterance meaning would come from findings showing that the interpretation of an utterance that is paired with separate visual signals (e.g., a head turn, or an an eyebrow raise) will be different from the interpretation of that utterance paired with the combination of those same signals. In terms of non-additive, Gestalt-like perception, we hypothesized that if signal *x* and signal *y* lead to a particular interpretation (or interpretations), signals *x* and* y* together lead to a different interpretation than either of the two alone, or the two single interpretations simply put together. This would not fit the additive model, as the interpretation of *x* + *y* cannot be derived from the interpretations of *x* or *y* in isolation.

## Results

### Response entropy across gestalts

**Figure 1:**
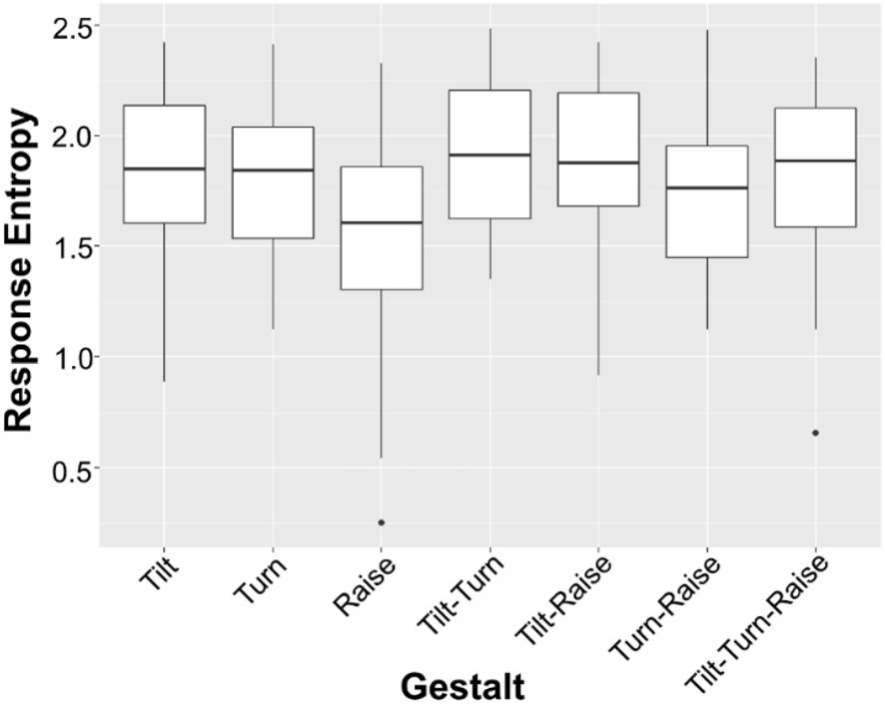
Response entropy across Gestalts. The y-axis provides response (Shannon) entropy, while the x-axis provides the Gestalts. Boxes depict the 25th and 75th percentiles (lower and upper hinges) and median (center line). Whiskers extend to the largest or smallest value no further than 1.5 times the interquartile range from the nearest hinge. Data beyond the whiskers are depicted as individual points.

Our first analyses assessed whether there was a similar distribution of variance across responses to the different Gestalts. In our analysis of response entropy, we find that entropy differs across the Gestalt categories *F*(6) = 2.67, *p* = 0.017. Mean entropy across the target Gestalts was 1.787 ± 0.42 (see Fig. 1 for response entropy per Gestalt). Tukey-adjusted post-hoc comparisons indicate that this is due to Brow Raises having lower entropy than Head Tilts (difference = 0.354, *p* = 0.049), Tilt + Turn (difference = 0.407, *p* = 0.013), and Tilt + Raise (difference = 0.368, *p* = 0.036). This indicates that, while most Gestalts elicit quite comparable degrees of response entropy, responses to Brow Raises are somewhat less variable.

Given the appearance of two outliers in Fig. 1, we carried out a post-hoc check of whether there are outlying datapoints with a strong influence on our model of response entropy. We calculated Cook’s Distance, which calculates how much model fit changes with the removal of each *i*_th_ datapoint, and removed datapoints with a Cook’s Distance greater than three times the mean Distance. This led to a removal of eleven datapoints (five of which were Brow Raise datapoints). The model without these datapoints also showed a significant difference in entropy across Gestalts, *F*(6) = 2.456, *p* = 0.027. However, Tukey-adjusted post-hoc comparisons revealed no significant contrasts between Gestalts. This suggests that, even when removing outliers, there is *some* evidence for a difference in response entropy.

### Responses to complex gestalts compared to pooled tier 1 single signal responses

Our next analyses assessed whether the distribution of responses to complex Gestalts (i.e., those with two or three visual signals) was more different from the responses to single signal simple Gestalts (i.e., those with only one visual signal) than what would be expected due to within-subject variability. This was done by calculating the absolute difference in response distributions, or *shift sum.* First, we calculated the shift sum between responses to the same Gestalt, but different question type (content-questions or personal questions), yielding a reference value of 0.53, indicating that this is the approximate magnitude of shift sum that is simply due to within-subject variation. Next, as our main analysis, we calculated shift sum values between complex and simple Gestalts. For this analysis, we specifically compared responses to complex Gestalts against the pooled responses to the constituent single signals (e.g., complex tilt-turn compared against pooled single tilt and single turn).

As expected, we found no evidence for question type contributing to shift sum values, χ^2^(3) = 3.068, *p* = 0.381. However, shift sum was found to differ across Gestalts, χ^2^(4) = 22.907, *p* < 0.001. Specifically, we found a larger shift in Tilt-Turn responses compared to Tilt-Raise values (*t* = 4.456). Additionally, we found that shift sum values significantly differed from our reference values (95% confidence interval for proportional difference from reference value: 0.068–0.204; *t* = 3.908).

**Figure 2:**
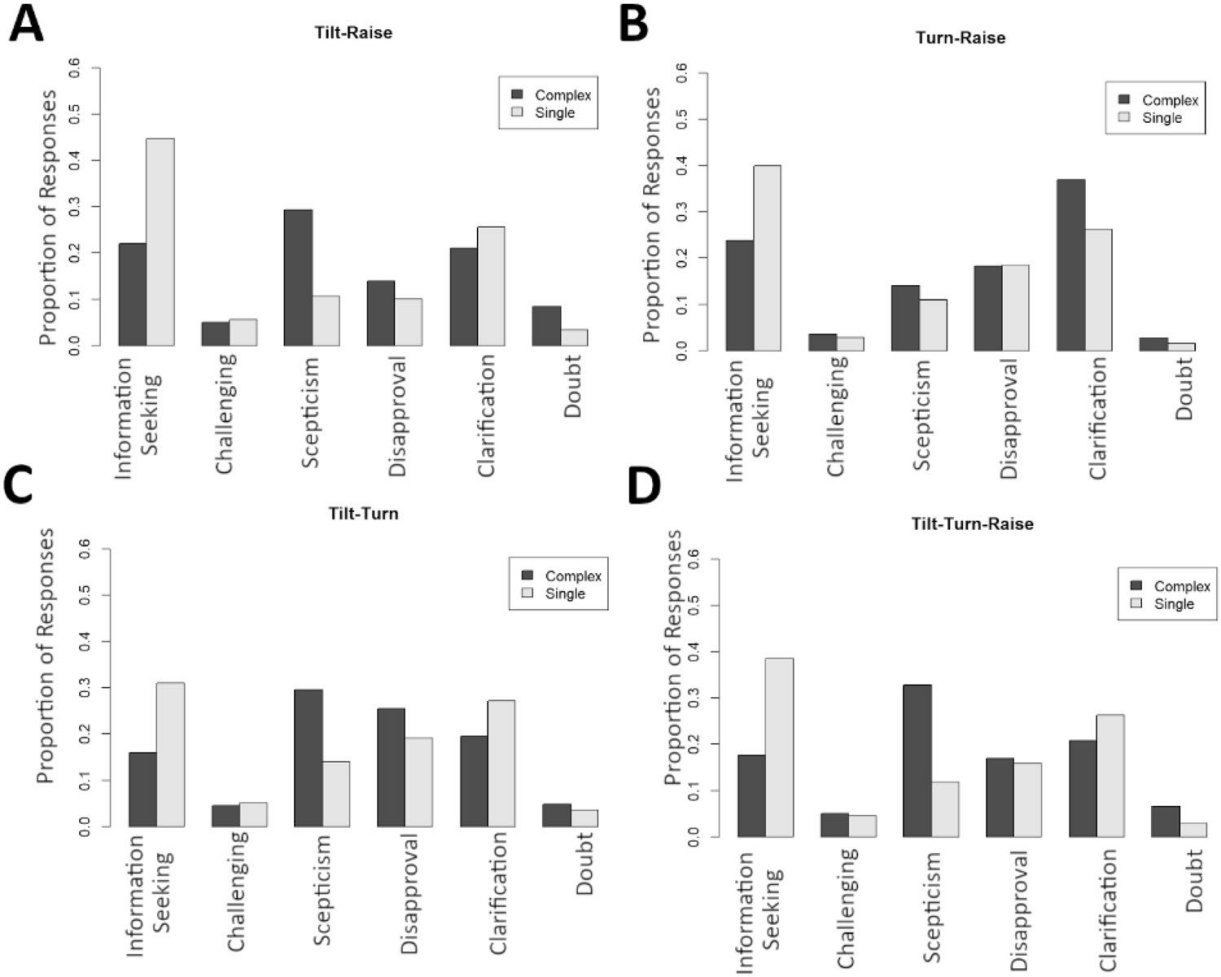
Overview of response distributions to each of the complex Gestalts compared to the pooled response distributions to the constituent signal Gestalts. Panel (**A**) shows Tilt-Raise, panel (**B**) shows Turn-Raise, panel (**C**) shows Tilt-Turn, and panel (**D**) shows Tilt-Turn-Raise. In all panels, proportion of responses (per category) is given on the y-axis, while the six response categories are displayed along the x-axis. Dark bars represent responses to the complex Gestalts, while light bars represent responses to the (pooled) single signal Gestalts.

Differences between responses to complex and pooled Tier 1 responses can also be seen by visualizing the response distributions (Fig. 2). Tilt-Raises (Fig. 2A) are primarily perceived as requests for *skepticism* (29% of responses) while the pooled responses for individual Tilts and individual Raises favor *information seeking* (45% of responses). Turn-Raises (Fig. 2B) are primarily perceived as requests for *clarification* (37% of responses), while the pooled responses for individual Turns and individual Raises favor *information seeking* (40% of responses). Tilt-Turns (Fig. 2C) are primarily perceived as expressions of *skepticism* (30% of responses) or *disapproval* (26%) while the pooled responses for individual Tilts and individual Turns favor *information seeking* (31% of responses) or requests for *clarification* (27%). Tilt-Turn-Raises (Fig. 2D) are primarily perceived as expressions of *skepticism* (33% of responses) while the pooled responses for individual Tilts, Turns and Raises favor *information seeking* (39% of responses).

### Responses to complex gestalts compared to single signal tier 1 responses

**Figure 3:**
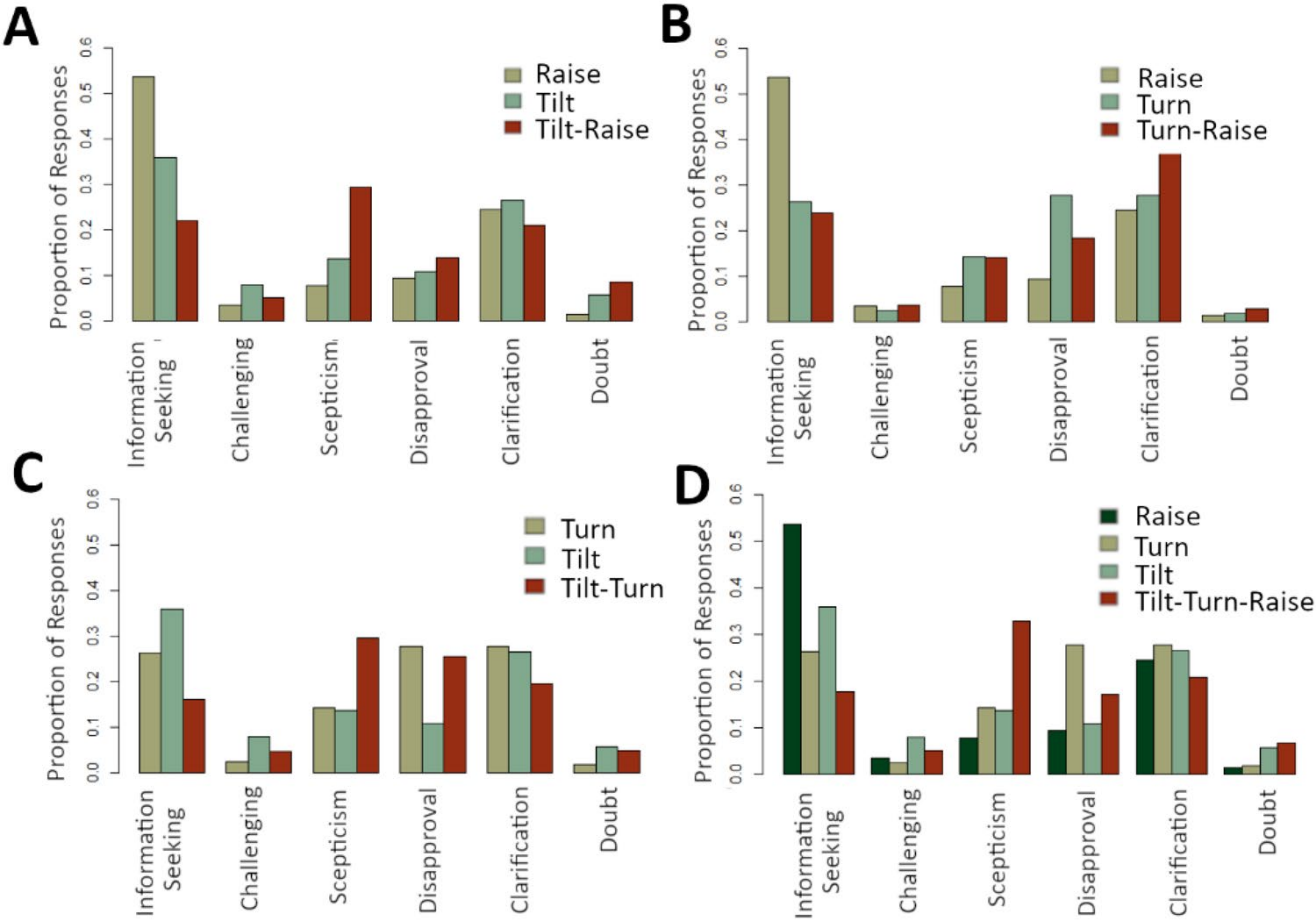
Overview of response distributions to each of the complex Gestalts compared to the response distributions to each of the constituent signal Gestalts. Panel (**A**) shows Tilt-Raise, panel (**B**) shows Turn-Raise, panel (**C**) shows Tilt-Turn, and panel (**D**) shows Tilt-Turn-Raise. In all panels, proportion of responses (i.e., number of responses to a particular Gestalt in each response category divided by total number of responses to the same Gestalt) is given on the y-axis, while the six response categories are displayed along the x-axis. The grouped bars represent the specific Gestalt (see legend in each panel for more information).

Using the same methodology as the previous analyses, we next compared responses to complex Gestalts against responses to each of the single signal Gestalts. In this analysis, we found evidence for a significant shift (i.e., greater than the reference value of 0.53) in responses for all of the comparisons, with p-values (after Holm’s correction) all < 0.001. An overview of the response distributions for each of these comparisons is provided in Fig. 3.

### Post-hoc: by-item analyses

While the above results indicate that participants generally responded differently to the complex Gestalts than to the individual signals, we wanted to specifically test whether responses to a particular utterance changed according to the Gestalt with which it was presented. Our first post-hoc analyses therefore carried out the same tests of complex vs pooled Tier 1 and complex vs single Tier 1 signals Gestalts as described above, but aggregating response distributions for each *item*, rather than for each participant.

We again found a significant difference in shift sum across our complex Gestalts χ^2^(3) = 20.403, *p* < 0.001. We also found that shift sum values in the by-item analysis significantly differed from our reference values (95% confidence interval for proportional difference from reference value: 0.278–0.384; *t* = 12.237).

In our by-item tests comparing complex Gestalts to the constituent single signal Gestalts, we again found evidence for a significant shift (i.e., greater than the reference value of 0.53) in responses for all of the comparisons, with p-values (after Holm’s correction) all < 0.001.

An overview of these results, including plots and test statistics, can be found on the OSF supplementary materials. An alternative visualization of these by-item shifts in response proportions can be seen in Supplementary Fig. 1.

### Post-hoc: within-tier comparison

Our second post-hoc test was designed to test whether the individual components (i.e., the Tier 1 single signals) differed from one another in terms of the response distributions. This would be evidence for the single signals being differentiable from one another, which is a requirement for compositionality. We found evidence for a difference in response distribution (i.e., greater than the reference value of 0.53) for all pairs of Tier 1 signals, with all p-values < 0.001 after Holm’s correction. This indicates that each individual signal elicited a different distribution of responses than the other individual signals.

### Post-hoc: within-gestalt vs out-of-gestalt comparison

**Figure 4:**
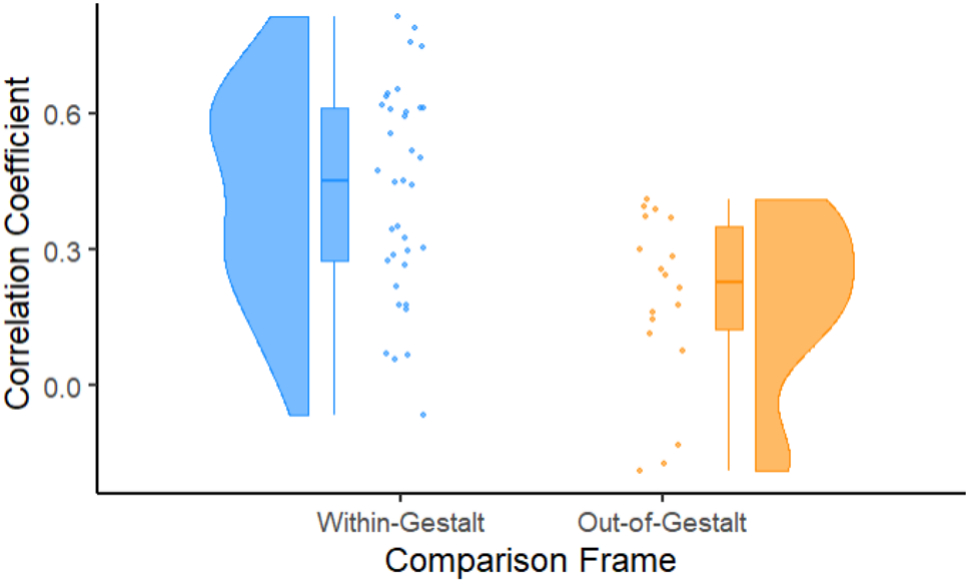
Correlations between responses to complex Gestalts and single signals as a function of whether the single signal is from the same complex Gestalt (Within-Gestalt) or not (Out-of-Gestalt). Pearson correlation coefficients are given on the y-axis, while Within-Gestalt and Out-of-Gestalt are indicated on the x-axis. Individual data points are given as dots, while colored curves represent the smooth probability distribution for the data and boxplots display the median (center line) and interquartile range (hinges). Whiskers on the boxplots extend to the furthest data point that is maximally 1.5 times the interquartile range away from the hinge.

Our final post-hoc test assessed whether responses to complex (Tier 2) Gestalts were more similar to within-Gestalt single signals compared to out-of-Gestalt single signals. This would be evidence for each single signal having a stable contribution to the more complex Gestalt, which is a key requirement for evidence of compositionality. We indeed found evidence for a difference in correlation coefficients, χ^2^(1) = 7.762, *p* = 0.005, with out-of-Gestalt responses showing 0.238 ± 0.08 lower coefficients (*t* = 3.096). See Fig. 4 for an overview of these results.

## Discussion

The aim of this study was to determine if facial and head signals that accompany utterances in face-to-face settings form part of the meaning speakers convey, thus suggesting that they form part of composite utterances and a compositional meaning system. In addition, we set out to test whether the individual signal meanings combined in an additive manner, or whether they can lead to a shift in meaning interpretation (i.e. combine in a non-additive, Gestalt-like manner).

We indeed found that participants interpreted the intention of the same spoken utterances differently depending on the (combination of) visual signals accompanying the speech. Specifically, responses to the same utterances accompanied by different single signals differed from one another, and the combinations of those signals led to different responses than when the speech was accompanied by the individual signals alone. This pattern of shifting interpretations occurred both at the level of participants (i.e., participants had preferred interpretations for each visual Gestalt) and, importantly, also at the level of individual utterances (i.e., interpretation of a given spoken utterance systematically changed according to the visual signals accompanying it). These findings directly replicate a pilot study using the same stimuli, which suggests that they are quite robust. The findings from this analysis underline the fact that participants’ responses were not simply determined by seeing the visual signal(s) themselves, but based on processing the utterances multimodally. The fact that the single visual signals, as well as the complex combinations, influenced pragmatic meaning interpretation, or intention attribution, of utterances when the spoken part remained entirely unchanged highlights the strong pragmatic impact conversational visual signals can have (which, based on the stimuli used here, in social interaction may even translate into changing the perception of an utterance from a neutral into a face threatening act^44^), thus highlighting the pragmatic power of conversational facial signals. This calls for a revision of the traditional notion of ‘speech acts’^45,46^ in favour of one that takes account of the multimodal nature of human language.

We also found evidence that the way a combination of two signals influenced interpretation of a spoken utterance could not be determined by the influence of the two signals when used separately or simply by putting them together. While individual signals elicit unique patterns of interpretation and have a statistically detectable influence on the interpretation of the whole, the intepretation of the whole cannot be predicted as an additive combination of these signals. These results provide first evidence, as far as we are aware, for compositional, as well as Gestalt-like perception of multimodal communicative behavior.

Our finding of intention attribution emerging from the specific combination of signals that constitute a multimodal utterance provides evidence for the multimodal compositionality of human language. Namely, that the meaning of the whole comes from a combination of individual parts, or “signals” in the case of our study, and that the meaning of the whole differs from the meaning of the parts. From a unimodal perspective, the non-additive, compositional nature of visual signals has previously been shown for facial expression^26,27^. Yet there was no evidence for whether multimodal language, the natural form of language use in face-to-face interaction for hearing individuals^15,18, 20, 47, 48^ also showed such non-additive compositionality. Our findings thus provide evidence that the pragmatic meaning of a spoken utterance, or the recognition of the social intentions they convey, emerges from a Gestalt-like interpretation of all available signals. Importantly, the fact that individual spoken utterances show a systematic shift in interpretation when paired with different combinations of visual signals suggests that the interpretation of pragmatic meaning is based on all available information. There is earlier evidence for visual and vocal signals jointly contributing to the expression of meaning, although many of these studies suggest an additive contribution of visual signals. For example, some research has suggested that particular configurations of facial signals (e.g., a frown, or a smile) contributes a specific emotion to the multimodally-perceived utterance^49^. While such stable contributions of visual signals may certainly exist, our findings show that such an additive interpretation cannot fully explain *pragmatic* interpretations of multimodal utterances. Specifically, the present study provides evidence that the interpretation of the individual signals does not directly predict the interpretation of the combinations of signals, which also supports the notion of non-additive, Gestalt-like interpretation. This non-additive contribution of multimodal signals is directly in line with a recent theoretical framework of face-to-face communication^19^, and thus provides, to the best our knowledge, the first evidence for such Gestalt-like multimodal utterance perception.

While our initial results showed evidence for non-additivity when combining visual signals, our post-hoc analyses set out to also test a basic, key requirement for compositionality: that individual components (i.e., visual signals, in the case of this study) have a consistent influence on the meaning of the whole. The meaning of the whole is more complex than the meaning of the parts, but there should be evidence that at least some parts push the meaning of the whole in a particular direction.

In addition to statistical evidence for consistent contributions of individual signals on the whole, we found that the interpretation of a combination of signals is closer to the interpretation of a single signal that also occurs in that combination, compared to the intepretation of an unrelated signal. This indicates that, within this Gestalt-like processing, there are consistent contributions from the individual signals (i.e., the parts, in Gestalt terms) on the interpretation of the whole. Together, our findings therefore support the notion that interpretation of multimodal communicative signals is Gestalt-like *and* compositional. The current findings therefore broaden the notion of compositionality, which is a central aspect in the conceptualization of human language^1,50^, providing evidence for the multimodal nature of compositionality.

While we see evidence for the meaning of the whole differing from the meaning of the parts, yet with consistent contributions of the parts, close examination of the results reveals differences in the strengths of these individual contributions. For example, head turns on their own may be somewhat ambiguous, as seen by the relatively equal distribution of responses in information seeking, disapproval, and clarification. Yet, when combined with eyebrow raises, the clarification interpretation stands out from the rest (see Fig. 3B–D). From the perspective of eyebrow raises, raises on their own are strongly associated with information seeking, and only secondarily with clarification. When combined with head turns, it is the secondary interpretation, clarification, that becomes favored. When eyebrow raises are combined with head tilts, we instead see a shift away from the favored interpretations of both individual signals, as the tilt-raise Gestalt is seen as skepticism. Indeed, when head tilts are combined with head turns, we again see a shift away from the favored interpretation of both individual signals. Similar to tilt-turn Gestalts, the tilt-turn-raise Gestalt is seen as skepticism, indicating that eyebrow raises had little influence on shifting the response distribution. The exact interpretation of a multimodal utterance will likely also depend on contextual factors, such as discourse history and social context. However, what these results indicate is that there is evidence for individual visual signals having both strong and weak effects on the meaning of the whole, even when the signal has a strong association with a particular interpretation on its own. These results thus further highlight that multimodal meaning is understood as both compositional and non-additive.

Our finding of a distribution of different interpretations to each Gestalt, rather than one highly favored interpretation, may at first seem to be at odds with the notion of compositionality, given that some authors only consider compositionality when meanings are highly consistent^28,30^. However, this may be due to the level of analysis. Namely, we are considering pragmatic interpretation, which may be more variable than, for example, emotion categorization. Indeed, others have argued that compositionality in human language means that, rather than individual parts contributing to a set meaning, they contribute to a range of possible of interpretations^51,52^, depending on other contextual factors. Therefore, individual signals in multimodal utterances may be interpreted differently to one another, and again differently depending on the overall composition that they are in. These signals may also contribute consistent influences on the interpretation of the whole. However, these interpretations are not absolute, single meanings, but rather a distribution of possibilities, with some interpretations being more strongly favored than others. For exampe, eyebrow raises were found to be strongly associated with information requests. Therefore, Gestalts that include eyebrow raises may be more strongly associated wtih information requests than Gestalts that do not include eyebrow raises. Yet information requests remain one *possible* interpretation within a larger distribution. How likely an addressee is to perceive an utterance as an information request, as opposed to another social intention, will depend on the other signals accompanying the eyebrow raise. As such, we argue that our study does provide evidence for Gestalt-like, compositional meaning in multimodal utterances, but that the notion of meaning in *compositionality* may be more flexible when addressing the pragmatics of multimodal language.

Our findings are also relevant for understanding the evolution of language. Specifically, the finding that multimodal signals are interpreted as one Gestalt meaning, resonates well with calls to study non-human communicative behaviors while taking into account the entire multimodal act^29,53, 54^. Our findings additionally provide an explanatory factor for the perceptual mechanisms underlying multimodal language use for models^55^ and experiments^56,57^ in human language evolution, which can inform models of the emergence of modern, multimodal language use. Specifically, our findings provide support for a highly integrated perceptual system^58,59^, wherein processing of each sensory modality is influenced by the rest^60–63^. The current work indicates that this integrated, holistic perception extends to the way communicative behavior is interpreted, possibly based on domain-general multisensory integration mechanisms^15^. This extension to communicative behavior can then inform theories of the evolution from perception and action to multimodally-constituted social interaction.

The present study also goes beyond earlier work focusing on the integration of congruent versus incongruent multimodal affective signals. For example, several studies have investigated how congruent presentation of emotion-signaling prosody and facial expression contribute to enhanced emotion recognition compared to unimodal signals^64–67^. Similarly, other studies show that particular facial signals contribute to better recognition of^68^, or are more or less compatible with, particular speech acts^36^. However, no previous studies have investigated how different combinations of (multimodal) signals jointly lead to the perception of new or different social intentions. Future work may build on our findings by additionally manipulating speech prosody or other non-linguistic cues, as has been done for emotion recognition, in order to further understand how these different multimodal components influence one another in interaction.

The current study further has implications for the development of social robots and virtual agents. First, our findings suggest that visual signals cannot simply be categorized for their pragmatic or affective meaning and added onto speech. Instead, the likely interpretation(s) of the full multimodal utterance will need to be determined, for example by collecting norming data on how similar linguistic-visual Gestalts are interpreted. This will be important for creating realistic social agents that are able to convey complex and subtle pragmatic meaning. Second, the same issue applies to decoding models that must interpret a human interactional partner’s utterances or accurately recognize their intention. The full multimodal array of signals must be taken together in order to come to an accurate interpretation of the user’s intended meaning. Such a holistic approach is similar to recent proposals for modality-invariant representation of multimodal sentiment analysis (e.g., Hazarika et al.^69^). However, our findings push the notion even further by suggesting that, rather than fusing modality-invariant representations, human-like perception should consider the modalities together from the beginning.

The current study provides strong empirical support for holistic, Gestalt-like interpretation of multimodal utterances due to its use of virtual avatars for controlled, yet realistic multimodal displays. The use of photorealistic avatars allowed us to create combinations of visual signals that smoothly integrate with spoken utterances. Our study is also robust in that it reports a direct replication of results from a pilot study, with pre-registered methods and hypotheses. This use of direct replication provides additional support for our statistical findings.

While our study provides evidence for the importance of investigating language understanding holistically, our study still isolates these multimodal signals from other factors that we expect to also contribute to pragmatic interpretation, such as prosody and other visual signals (e.g. hand gestures and body shifts). Also, the larger interactional embedding of an utterance, which includes discourse context, learned idiosyncrasies of the speaker, and the observer’s own affective state all likely shape utterance interpretation^19^. Finally, the present study only assessed how the general population responded to multimodal Gestalts. Future research may also determine whether these patterns of results extend to neurodivergent groups that may have different social and perceptual styles, such as autistic or schizophrenic individuals. It is also important to note that while we describe the six response options as relating to a particular intention (e.g., disagreement, doubt), this study was not meant to suggest that a particular response option maps one-to-one to a particular intention. For example, disagreeing with someone may also be a way to signal disapproval, rather than (or in addition to) issuing a challenge. In our current study, we use the intention category labels (e.g., disapproving, challenging) to more succinctly describe what we take to be the primary intention that is perceived by the participants in our study. Returning to the example of disagreeing, we assume that if a participant perceived the avatar as disagreeing in order to signal disapproval, that they would select the response option for disapproval rather than disagreement. While it is not possible to know with certainty whether participants always followed this strategy, the interpretations of our findings still hold, even if we take away the intention labels and only consider the full-sentence response options. Still, future research could benefit from further examining the relationship between the categorical labels of intentions (e.g., “disagreeing”) and the more fine-grained intentions behind the action (e.g., simply disagreeing, or disagreeing to express disapproval).

One unexpected finding that differed from our initial pilot study was the differing response entropy across visual Gestalts. Even after removing outlier datapoints, we still see a general statistical difference across the visual conditions, although post-hoc contrasts show no specific differences. This finding suggests that brow raises in particular elicit somewhat less variable responses than the other Gestalts. However, that brow raises were the only signals to show a significant difference, and the difference was having lower entropy than others, also suggests that this is unlikely to have impacted our main analyses. Specifically, the results indicate that most Gestalts and visual signals actually elicit a similar level of response entropy, and importantly, none of the Gestalts elicit response entropy that is significantly *higher* than the rest which would be most likely to confound results.

Our study provides evidence for non-additive, compositional, Gestalt-like interpretation of the pragmatic meaning (i.e., social intention, or social action) of multimodal language. This study therefore has implications for models of human language and cognition, language evolution, and the generation of artificial conversational agents.

## Methods

### Pre-registration and pilot study

Current analyses are based on a pilot study using the same materials. The preregistration for the current study, based on this pilot study, can be found here: https://aspredicted.org/blind.php?x=BZ3_MDM.

### Participants and ethics

Ten males and fourteen females (Mean age = 28.3 ± 8 years), all native Dutch speakers, born and currently living in the Netherlands, completed the experiment. All participants used a computer to complete the task (i.e., no smartphones or tablets). Participants were recruited using the online Prolific platform. Online participants were chosen in order to sample from the wider public rather than just the student population.

The study was approved by the Ethics Committee Faculty of Social Sciences at Radboud University Nijmegen and all procedures to be performed are in accordance with the Declaration of Helsinki (World Medical Association, 2013). All participants were required to provide informed consent before participating in the study. Participants were compensated with 9€ upon completing the experiment.

### Materials

**Figure 5:**
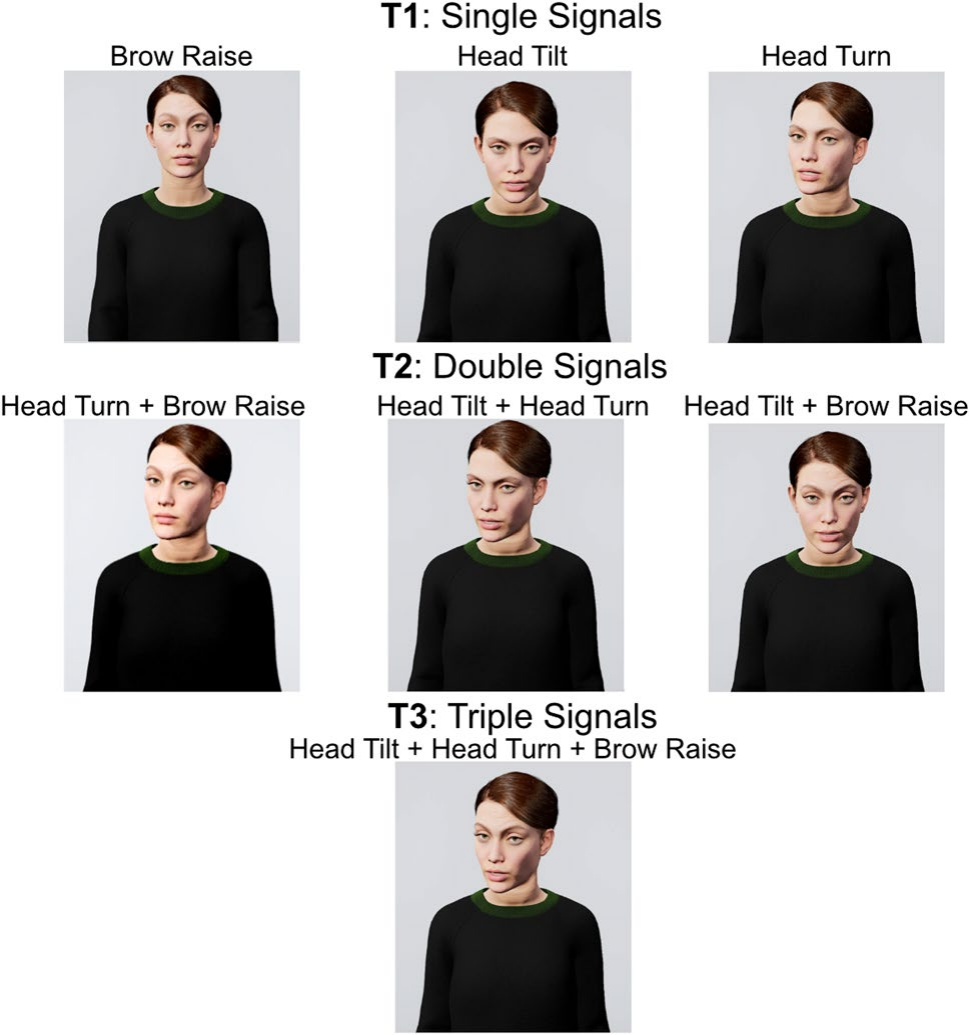
Overview of avatar appearance and visual Gestalts. Each panel depicts one of the visual Gestalts, with all signals at their maximum amplitude (e.g., eyebrow raise amplitude, extent of head turn). The rows of panels represent the Gestalt grouping Tiers, with Tier 1 showing single signals, Tier 2 showing double signals, and Tier 3 showing the full, three-signal composition.

Stimuli consisted of videos of a virtual avatar uttering questions while producing various visual signals (see Fig. 5). Virtual avatars were used in order to provide a realistic, dynamic agent while still maintaining experimental control and consistency across conditions^70,71^. Audio recordings were spoken questions (n = 252), uttered by a female native Dutch speaker, and were a mixture of personal (e.g., “Do you know anyone who works as a baker?”) or general factual questions (e.g., “Does sushi originally come from Japan?”). Visual signals were manually animated in Maya^72^ on a MetaHuman^73^ skeleton rig, resulting in photorealistic avatar renditions. We created one neutral animation, that only contained “ambient motion” animations (described below), and seven (7) target animations (i.e., Gestalts) which consisted of three single signals (head turn, head tilt, eyebrow raise) and the four possible combinations of these signals (head turn + head tilt, head turn + eyebrow raise, head tilt + eyebrow raise, head turn + head tilt + eyebrow raise). See Fig. 5 for an overview of these signals, and see Supplementary Material for example videos of each. These three signals were chosen after observing them being used both in isolation, and in the two and three signal combinations in a corpus of face-to-face conversation betweens Dutch speakers^37,38^. We additionally created five filler animations that were designed to distract participants from focusing too much on the target animations. The filler animations were signals that also frequently accompany questions in casual face-to-face conversations^24,37, 38^: palm-up open-hand gesture, lateral hand gesture, eye squint, eyebrow frown, and nose wrinkle. For all animations, besides the primary signal (e.g., head turn, hand gesture), we also included three forms of ambient motion in order to make the avatar more lifelike^74^. These ambient motion animations were eye blinks, torso sway, and ambient gaze. Eye blinks occurred at random times throughout an utterances, and were always short in duration in order to avoid potentially pragmatic effects of long blinks^75^. Torso sway was created by generating random sinusoid ranging from − 0.5 to + 0.5 and applying this to the x, y, and z rotation parameters of the avatar spine. Ambient gaze added a slow, random drift to the eyes, and was automatically generated using Jali^76^. In order to combine the audio recordings with the virtual avatar in a realistic manner, we used the Jali^76^ software to automatically generate language-accurate, synchronised lip movements for each of the audio recordings. Audio, lip-movement, and visual signals were combined and rendered in Unreal Engine (v.4.26; Epic Games, 2020).

We created eight stimulus lists, each containing 24 utterances paired with each of the seven Gestalts as well as the neutral animation, totaling 192 “target” videos in each list. The separate lists ensured that, across the entire set of eight lists, each spoken utterance was paired with each Gestalt, without individual participants hearing the same question more than once. We additionally created 35 filler videos, utilizing the five filler animations. This led to a total of 227 items in each stimulus list. Within each list, each Gestalt and filler animation had an equal number of personal and general Question Types.

The intentions described, and that served as the response options for the participants were: “The speaker is asking for information” [de avatar vraagt om informatie] (intention: seeking information), “the speaker is disagreeing” [de avatar gaat er tegenin] (intention: challenging), “the speaker is skeptical” [de avatar is sceptisch] (intention: expressing skepticism), “the speaker is disapproving” [de avatar is afkeurend] (intention: expressing disapproval), “the speaker is asking for clarification” [de avatar vraagt om clarificatie] (intention: clarification request), “the speaker is doubting” (intention: expressing doubt). These six categories are based on the most frequent question types observed in an earlier corpus study of Dutch question-response pairs^77^, namely Information requests, understanding checks, and stance/sentiment questions. These three base categories were further subdivided into the six intention categories used in the present study based on early pilot testing that suggested that more categories allowed more meaningful distinctions to be made. While additional categories, such as irony or persuasion, would certainly be relevant and interesting, we chose to also limit the number of possible answer choices to ensure that participants were not overwhelmed by the number of possible answers. Given that these intentions were found in the same corpus in which we observed the visual Gestalts used in the present study, these intentions may also be more likely to be associated with the visual Gestalts than other potential question intentions. The six categories were also meant to ensure that not all response options carried a negative tone. Specifically, disapproval, skepticism, and challenging intentions can be seen as potentially negative. Information seeking and clarification are more positive, or at least not necessarily negative. Doubt can perhaps be seen as in between these two sides, as doubt implies that the speaker may have conflicting opinions or evidence and is thus uncertain, whereas skepticism implies that the speaker is leaning towards a particular answer but is asking for evidence to sway their opinion. For example, one could utter “does sushi come from Japan originally?”, and they would be in doubt if they are uncertain if sushi comes from China or from Japan. On the other hand, the same utterance could be produced skeptically, if the speaker believes that sushi comes from China, but they are inviting the addressee to correct them. To further clarify differences between the intention categories, the same utterance could be challenging if the speaker is quite certain that sushi comes from China, and they are using the question to challenge the belief of the addressee, who may have just stated that sushi comes from Japan.

### Procedure

The experiment was designed and run using the Gorilla^78^ online experiment platform. Participants were first briefed on the possible intentions that a question can perform, which were framed as intentions that one can express, and did not make any reference to multimodal signals. See Appendix I (*Supplementary Materials*) for the full set of instructions.

Before the experiment started, participants performed two short tasks designed to check audio quality and to ensure participants could understand the speech. First, there was a short sound check to ensure that the audio was working, which also allowed participants to adjust their volume before continuing. Next, participants heard two questions, spoken by a native Dutch speaker, and were asked to type an answer in response. These comprehension questions were simple, common knowledge questions. First, participants heard “Wat is het vijfde letter van het alfabet?” [“What is the fifth letter of the alphabet?”]. Next, participants heard “Ik word gebruikt om te kunnen schrijven samen met papier. Wat ben ik?” [I’m used to write, together with paper. What am I?”]. Incorrect answers to either of these questions was used as an exclusion criterion based on a lack of Dutch language proficiency.

Participants then began the experiment. Each trial started with a fixation cross lasting 400 ms. The video then played, followed by a response screen. On the response screen, the same six response options listed above were provided, and participants selected their response using the 1–6 numbers on the keyboard (the order of the response options was not randomized in order to not force participants to read the relatively long list of response options after every trial. After a response was recorded, a blank screen appeared for 500 ms. The experiment was divided into four blocks, giving participants the opportunity to take a short break between each block.

### Analysis: response entropy

As a first step, we calculated whether participants responses are more or less variable in some Gestalts than others. This allows us to determine if there is evidence for some Gestalts being less consistently categorized than others and serves as a first check that variance across the Gestalts is relatively normally distributed. To this end, we calculated Shannon entropy for each participant’s distribution of responses to each Gestalt. This effectively provides a metric of how variable the response distribution was, given the number of possible responses. We then performed a standard analysis of variance (ANOVA) to test whether entropy differed across the different Gestalts.

### Analysis: calculating shift sum values

Proportion of responses is calculated, per participant, based on the number of responses in each intention category, for each Gestalt. Each participant saw each Gestalt 24 times (with 24 different spoken utterances), so the number of responses per category is divided by 24 to get the *proportion of responses* for each Gestalt. In order to statistically test whether there is a shift in the distribution of responses, we utilize absolute differences in response portions between either (Analysis 1) the pooled tier-1 Gestalts and tier-2 + Gestalts, or (Analysis 2) between responses to each of the individual signals and the tier-2 + Gestalts. We then take the sum of these differences, and test whether this difference is significantly different from what we would expect by chance. See subsection “Analysis: calculating reference value” for more information on how we calculate this chance-level reference value.

Calculating the actual proportional response difference is done grouped by participant, Question Type, and Gestalt. See Table 1 for a toy example of how the shift sum value can be calculated in one participant, question type, Gestalt case. Note that, in Table 1, we show 3 response categories, rather than 6, for simplicity of the example.

**Table 1:** Illustration of a hypothetical example of the *shift sum* calculation (based on three response options only, for sake of simplicity).

	Proportion of responses in intention category 1	Proportion of responses in intention category 2	Proportion of responses in intention category 3
Tier 1 values	0.50	0.15	0.35
Tier 2 + values	0.20	0.50	0.30
Difference	0.30	0.35	0.05

In the example in Table 1, the sum of the differences is 0.70 This shift sum value gives us a quantification of the shift in categorization, utilizing the full response distribution. Finally, we subtract the *reference value* (described below) from all shift sum values in our dataset. This allows us to test whether the shift sum values are significantly greater than the reference value (i.e., whether there is a greater shift in response distributions than would be expected by chance).

We then analyze these data by building a mixed linear regression model with shift sum as the predictor, Gestalt and Question Type as fixed effects, and participant ID as a random effect. The fixed effects indicate whether the perceptual “shift” differs on these factors (e.g., to determine if there is a greater shift in some Gestalts than others), while the intercept of the model tells us whether the shift is significantly different from zero (i.e., the reference value that was subtracted from the data). This approach allows us to model all effects of interest in one model, and provides us a statistical test and estimation of the true value of shift sum while accounting for other factors. We use likelihood ratio tests to incrementally test whether Question Type and Gestalt explain a substantial amount of the variance, beyond the base model (i.e., an intercept-only model with only the random term and the dependent variable). Our primary hypothesis test is based on whether the 95% confidence interval of the model intercept is greater than zero.

The above model-building approach is done in two separate analyses. In Analysis 1, we use the pooled Tier 1 signals compared to the Gestalt. In other words, we compare the distribution of responses to (for example) head turn + eyebrow raise to the distribution of head turn and eyebrow raise when pooled together and treated as one Gestalt. This analysis allows us to test specifically whether categorization of the Gestalt is similar to a linear (i.e., additive) combination of the responses to the constituent signals. In Analysis 2, we calculate separate shift sum values for each single signal-Gestalt pairing. In other words, we calculate the shift sum for head turn + eyebrow raise when compared to head turn, and compared to eyebrow raise. This analysis allows us to test whether categorization of the Gestalt interpretation is based on a single, dominant constituent signal. The two analyses thus provide complementary tests that allow us to determine whether the perception of Gestalts is linearly related to (i.e., directly predictable by) perception of the individual signals that constitute the Gestalt. We accounted for the increased Type I error rate associated with these multiple tests by apply Holm’s correction to the resulting p-values.

### Analysis: calculating reference value

In order to assess whether any shift in response distributions is greater than what would be expected by normal variance in participant responses, we calculated a reference value. We calculated our reference value based on the variation in responses to the same Tier 1 single signals across different question types. We did this by splitting the questions based on their *answer point* (i.e., whether the crucial information in the question came relatively earlier or later in the utterance)*,* a categorization that was created for another study using the same audio materials, but bears no relevance for the current study. This split in the questions thus provides us with two essentially equal groups of questions, where there is no hypothesized difference in terms of the expected responses. Specifically, we calculated the *shift sum* value based on the distribution of responses to head tilts, head turns, and eyebrow frowns performed with early answer-point questions compared to the responses to the same signal performed with late answer-point questions. This was done given that we did not hypothesize there to be a systematic shift in responses based on the type of question. Instead, we expected the difference between early and late answer-point questions to represent the natural variation in responses to the same visual signals performed with different questions. Finding shift sum values that significantly differ from this reference value, when comparing Gestalts to single signals, would therefore indicate that participants are perceiving the Gestalts differently than the single signals. Our reference value was found to be 0.527, indicating that internal variation (i.e., between early and late answer point questions) is approximately 26.35% change in response proportions.

### Post-hoc by-item analysis

As an additional analysis, we performed an alternative test of the *shift sum* values discussed above. The purpose of this analysis was to specifically test whether the interpretation of a given utterance differs according to the visual signals that it is paired with. Specifically, the analysis described above aggregates values for each participant, effectively testing whether participants’ responses to, for example, head turns and their response to brow raises differed from their responses to head turn + brow raise. Given that we are interested in how the interpretation of an utterance can change based on co-occurring visual signals, we also re-formatted the data to aggregate *across* participants, giving us a response distribution for each spoken utterance, in each visual signal/Gestalt condition. This addition is important because it more directly tests if the interpretation of an utterance paired with head turn, and the interpretation of the same utterance paired with brow raise, are different from the interpretation of the same utterance paired with head turn + brow raise. We then carried out the same analyses as described above, using this by-item dataset.

### Post-hoc within-tier comparison

Another important aspect of compositionality is that each component of the whole has a unique, identifiable meaning on its own. To test whether this is the case in our data, we ran an additional comparison between the Tier 1 signals. If the response distributions for these single signals differs from one another (as well as their combined interpretation from the single ones), this would be evidence in favour of a multimodal compositionality interpretation. For this analysis, we calculated *shift sum* values between each pair of Tier 1 signals (Tilt vs Turn, Tilt vs Raise, Turn vs Raise) and again tested these values against the reference point described above (0.527) using the Wilcoxon Rank Sum test. Similar to our Analysis 2 pipeline described above, we accounted for the increase in Type I error from multiple tests by using Holm’s correction.

### Post-hoc within- vs. out-of-gestalt comparison

Finally, in the formal definition of compositionality, each individual component must have a consistent contribution to the meaning of the whole. In some cases, the combination of signals leading to a unique interpretation has been reported, but without evidence of those signals showing a consistent contribution to the new interpretation (sometimes referred to as *componentiality, e.g.* Oña et al., 2019)*.* We therefore aimed to determine whether human multimodal utterances show evidence for compositionality in this regard also. For this analysis, we assessed whether response proportions to Gestalts are more similar to response proportions to single signals that come from the same Gestalt (e.g., Tilt-Turn compared to Turn) compared to single signals that are not from the same Gestalt (e.g., Tilt-Turn compared to Raise). Specifically, we calculated Pearson’s correlation coefficient between each pair of single signal and Gestalt, using the response proportions in each response category. We then used a linear mixed effects model to test whether the correlation coefficients for the within-Gestalt comparisons were greater than the coefficients for the out-of-Gestalt comparisons. These models used the coefficient as dependent variable, and within- vs out-of-Gestalt as independent variable. As each comparison had six correlation coefficients (one for each response category) we also included random intercepts for “Gestalt comparison” (random slopes resulted in singular model fit). We again compared this full model against a “null” model that did not include the main independent variable, using a likelihood ratio test of model comparison, to assess whether including within- vs out-of-Gestalt explained a significant portion of the variance. If the within-Gestalt comparisons show higher correlation coefficients, this would be evidence in favor of a compositional interpretation.

### Supplementary Information

Supplementary Information.

## Data Availability

Materials and a full report of the results from the pilot study, as well as from the current study, can be found on the Open Science Framework: https://osf.io/z4q2j/.
