# Supplementary material for: Conversational facial signals combine into compositional meanings that change the interpretation of speaker intentions
Source: Sci Rep. 2024 Jan 27;14:2286. doi: 10.1038/s41598-024-52589-0 (PMC10821935; doi:10.1038/s41598-024-52589-0)
Supplement: Supplementary file 1 — Supplementary Information. [file 41598_2024_52589_MOESM1_ESM.docx]

Supplementary Material for **Conversational Facial Expressions are Compositional and** **Elicit Gestalt-like Interpretation of Speaker Intentions**

James P. Trujillo^1,2^ & Judith Holler^1,2^

^1^ Max Planck Institute for Psycholinguistics, Nijmegen, The Netherlands

^2^ Donders Institute for Brain, Cognition, and Behaviour, Nijmegen, The Netherlands


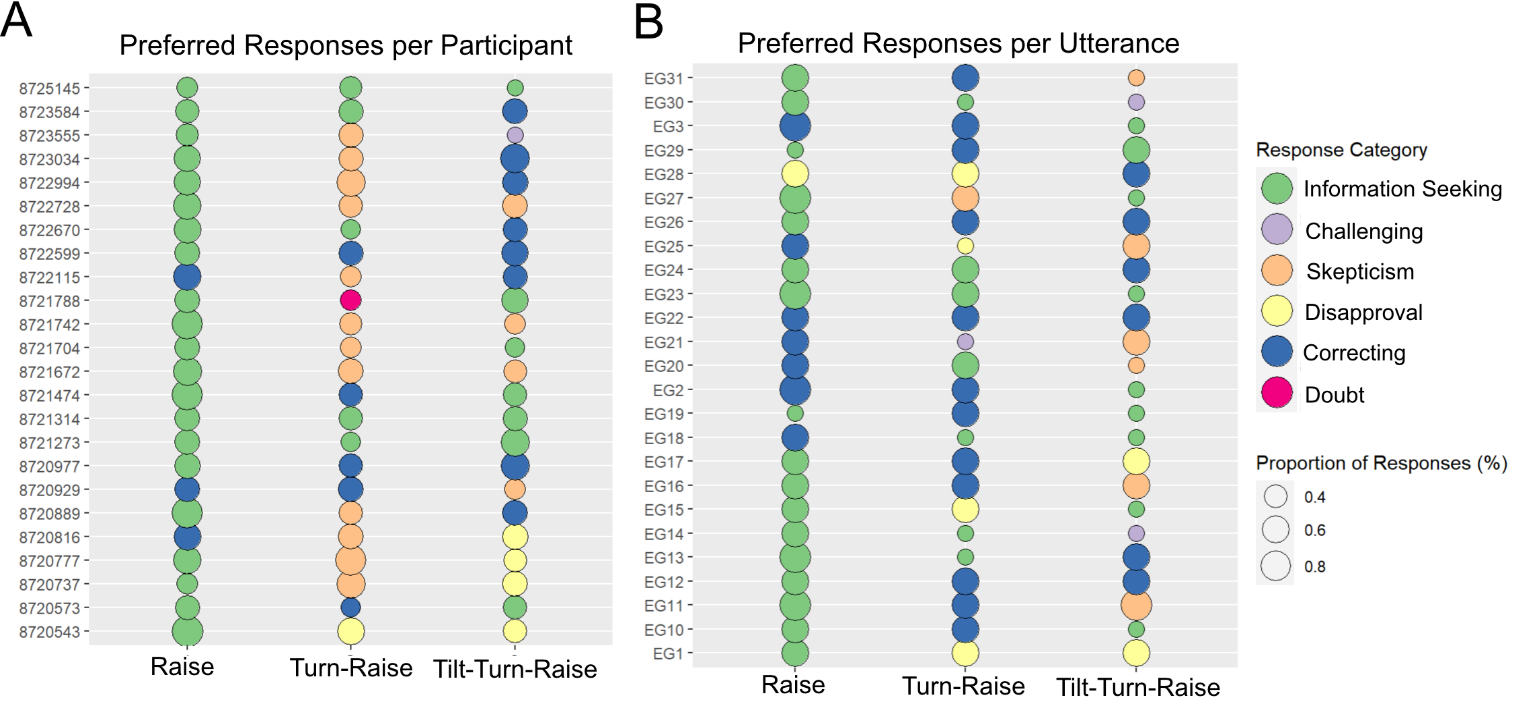


Supplementary Figure 1. Balloon Plots of preferred response category for each participant, and for each utterance, when paired with a single, double, or triple-signal visual Gestalt. In panel **A**, individual participants are given along the y-axis. In panel **B** individual items are given along the y-axis. Note that, for the sake brevity, we include only the first 25 utterances in this visualization (Note that all utterances were used in the actual statistical tests). In both panels, the 3 tiers of visual Gestalts (i.e., single, double, triple) are given on the x-axis. For this visualization, we used Raise (tier 1), Turn-Raise (tier 2), and Tilt-Turn-Raise (tier 3). Preferred response category is indicated by the color of the circle, while the size of the circle indicates the proportion of responses given in the preferred category. The plot visualizes that while there are preferred interpretations of each visual Gestalt when summarized across items (panel A), these preferences are also influenced by the speech portion of the multimodal Gestalt and are much more variable at the item level (panel B).

Appendix I.

**Experiment instructions (English translation and original Dutch)**

[People can ask a question for different reasons. Sometimes you just want to know something, but sometimes you use a question to show disapproval, or that you are doubting about something, for example if you know something isn’t correct. You can also ask a question to clarify a miscommunication, or if you don’t agree with the other person and want to challenge what they said.

During this experiment we ask you to watch short video clips of an avatar (virtual person) who asks short questions. How would you interpret the question?

Your task is to watch the videos and indicate, after each video, what you think the stance or intention of the avatar is. You can choose from six (6) different options:

1. The speaker is asking for information
2. the speaker is disagreeing
3. the speaker is skeptical
4. the speaker is doubting
5. the speaker is asking for clarification
6. the speaker is disapproving

There are no right or wrong answers. You don’t have to use all categories. Indicate your choice by typing the number (1,2,3,4,5,6) on the keyboard. ]

Mensen kunnen een vraag stellen met verschillende redenen. Soms wil je gewoon iets weten, maar soms laat je daarmee zien dat je iets afkeurt, of dat je ergens over twijfelt, bijvoorbeeld als je weet dat iets niet klopt. Je kan ook een vraag stellen als je een miscommunitie wil ophelderen, of als je het niet eens bent met de ander persoon en je wil er tegenin gaan.

Tijdens dit experiment vragen we je om te kijken naar korte filmpjes van een avatar (virtueel persoon) die korten vragen stelt. Hoe zou je de vraag interpreteren?

Je taak is om te kijken naar de filmpjes en aan te geven, bij elk filmpje, wat jij denkt de stemming of intentie van de avatar is. Je mag kiezen uit zes (6) verschillende opties:

1) de avatar vraagt om informatie,

2) de avatar gaat er tegenin,

3) de avatar is sceptisch,

4) de avatar twijfelt,

5) de avatar vraagt om opheldering,

6) de avatar is afkeurend.

Er zijn geen goede of foute antwoorden. Je hoeft niet alle categorieën te gebruiken. Je geeft je keuze aan door op de cijfer (1,2,3,4,5, of 6) op de toetsenbord te tikken.
